# Insoluble Aβ overexpression in an *App* knock-in mouse model alters microstructure and gamma oscillations in the prefrontal cortex, affecting anxiety-related behaviours

**DOI:** 10.1242/dmm.040550

**Published:** 2019-09-01

**Authors:** Eleftheria Pervolaraki, Stephen P. Hall, Denise Foresteire, Takashi Saito, Takaomi C. Saido, Miles A. Whittington, Colin Lever, James Dachtler

**Affiliations:** 1 School of Biomedical Sciences, University of Leeds, Leeds, LS2 9JT, UK; 2 Hull York Medical School, University of York, Heslington, YO10 5DD, UK; 3 Department of Psychology, Durham University, South Road, Durham, DH1 3LE, UK; 4 Laboratory for Proteolytic Neuroscience, RIKEN Center for Brain Science, Wako-shi, Saitama, Japan

**Keywords:** Alzheimer's disease, *App^NL-G-F^* knock-in mice, Anxiety, Prefrontal cortex, Gamma oscillations, Social

## Abstract

We studied a new amyloid-beta precursor protein (*App*) knock-in mouse model of Alzheimer's disease (*App^NL-G-F^*), containing the Swedish KM670/671NL mutation, the Iberian I716F mutation and the Artic E693G mutation, which generates elevated levels of amyloid beta (Aβ)_40_ and Aβ_42_ without the confounds associated with APP overexpression. This enabled us to assess changes in anxiety-related and social behaviours, and neural alterations potentially underlying such changes, driven specifically by Aβ accumulation. *App^NL-G-F^* knock-in mice exhibited subtle deficits in tasks assessing social olfaction, but not in social motivation tasks. In anxiety-assessing tasks, *App^NL-G-F^* knock-in mice exhibited: (1) increased thigmotaxis in the open field (OF), yet; (2) reduced closed-arm, and increased open-arm, time in the elevated plus maze (EPM). Their ostensibly anxiogenic OF profile, yet ostensibly anxiolytic EPM profile, could hint at altered cortical mechanisms affecting decision-making (e.g. ‘disinhibition’), rather than simple core deficits in emotional motivation. Consistent with this possibility, alterations in microstructure, glutamatergic-dependent gamma oscillations and glutamatergic gene expression were all observed in the prefrontal cortex, but not the amygdala, of *App^NL-G-F^* knock-in mice. Thus, insoluble Aβ overexpression drives prefrontal cortical alterations, potentially underlying changes in social and anxiety-related behavioural tasks.

This article has an associated First Person interview with the first author of the paper.

## Abstract (precis)

**Summary:** Aβ overexpression in a second-generation Alzheimer's disease model results in alterations in anxiety-type behaviours, as well as gamma oscillations, neural organisation and *Grin2b* expression in the prefrontal cortex.

## INTRODUCTION

Alzheimer's disease (AD) is classically associated with declining cognitive function (Scheltens et al., 2016). However, this is only one aspect of the behavioural changes associated with AD. Other behavioural changes include reduced social engagement and increased anxiety. Although social aspects of AD have remained underexplored, social withdrawal is present up to 5 years prior to a clinical cognitive diagnosis (Jost and Grossberg, 1995). AD patients with larger social networks (the number of people with which one has meaningful contact with) have slower cognitive decline, compared to AD patients with small social networks (Bennett et al., 2006). Social factors may modulate the rate of disease pathology and cognitive decline but, crucially, also the chance of developing AD (Kuiper et al., 2015). Studies have found that, for elderly people who identify as lonely, the risk of developing AD was nearly doubled (Wilson et al., 2007). Recent evidence from the Lancet Commission Report highlights that social isolation constitutes 2.3% of the total risk factors for developing AD (Livingston et al., 2017). Thus, social factors not only modulate the risk of developing dementia, but also disease progression. Together, it can be inferred that changes in social motivation of the individual, as a result of AD pathology, may be a factor in disease progression.

Anxiety in AD is relatively common, with up to 71% of patients reporting anxiety concerns (Ferretti et al., 2001; Teri et al., 1999). Up to 6% had anxiety that reached the diagnostic criteria of generalised anxiety disorder of the Diagnostic and Statistical Manual of Mental Disorders (Ferretti et al., 2001). Anxiety behaviours may also predict conversion to AD. Mild cognitive impairment (MCI) is often the precursor condition to AD. In total, 83.3% of MCI patients that also exhibited anxiety symptoms converted to AD within a 3-year follow-up period compared to 40.9% of persons with MCI but without anxiety (Palmer et al., 2007). This and other studies (Gallagher et al., 2011; Li and Li, 2018; Pietrzak et al., 2015) suggest that anxiety is associated with early phases of AD. Neurodegeneration in early AD may explain the increase in anxiety. The presence of anxiety is associated with abnormal cerebrospinal fluid (CSF) Aβ_42_ and tau in MCI patients (Ramakers et al., 2013), and thus may reflect underlying pathology. In summary, social-related and anxiety-related changes in AD can occur early in, and predict, disease progression.

The AD-affected neural regions relevant to changes in anxiety and social behaviours remain unclear, but candidate regions of interest (ROIs) for the present study were the hippocampus, amygdala and prefrontal cortex. Our rationale for these ROIs was twofold. First, these regions have been consistently linked to anxiety and social behaviours (e.g. hippocampus: Bannerman et al., 2004; Hitti and Siegelbaum, 2014; Okuyama et al., 2016; amygdala: Shin and Liberzon, 2010; prefrontal cortex: Cao et al., 2018). Second, pathology in these regions can occur early in AD and predict MCI-to-AD conversion (e.g. hippocampus: Devanand et al., 2007; Liu et al., 2010; amygdala: Liu et al., 2010; Poulin et al., 2011; prefrontal cortex: Okello et al., 2009; Plant et al., 2010; Tondelli et al., 2012). Interestingly, in Okello et al. (2009), the anterior cingulate cortex (ACC) had the highest amyloid-beta (Aβ) load of six ROIs in MCI patients relative to controls, and a higher anterior cingulate Aβ load predicted faster conversion from MCI to AD.

Currently, we have few insights into the biological mechanisms underpinning anxiety and social withdrawal in AD, highlighting the need to find AD mice that model these behaviours.

A biological mechanism that has been associated with dysfunction in AD are gamma oscillations. Gamma disruption has been demonstrated in other AD mouse models, such as the APP/PS1 model (Klein et al., 2016), the TAS10 overexpression model (Driver et al., 2007) and in the entorhinal cortex of *App^NL-G-F^* knock-in (KI) mice (Nakazono et al., 2017), and these impairments occur relatively early in Aβ-driven pathological processes. Gamma oscillations have been widely associated with roles in learning and memory (Tallon-Baudry et al., 1998), and attention (Fries et al., 2001), whilst coherence between brain regions has been demonstrated to facilitate information transfer (Buzsaki and Wang, 2012). Specifically in the prefrontal cortex, gamma oscillations have been shown to be important for social behaviour (Cao et al., 2018) and anxiety behaviours (Dzirasa et al., 2011; Stujenske et al., 2014). Thus, gamma oscillations represent a useful target for exploring network-level sequelae of AD-related pathology, relevant to social and anxiety-related behaviour. N-methyl-D-aspartate (NMDA) receptors (heteromeric complexes formed from GluN1 and GluN2A-GluN2D) are necessary for excitatory glutamatergic neurotransmission. NMDA dysfunction is well established in AD, such that one of the few drugs to ameliorate the symptoms of AD is the NMDA antagonist memantine (Lipton, 2006). Post-mortem studies have indicated that GluN1/GluN2B heterodimer expression significantly decreases with increasing AD pathology, whereas GluN1/GluN2A receptors (another form of the fast NMDA receptor) remain unchanged (Mishizen-Eberz et al., 2004). Thus, the contribution of NMDA receptors to normal synaptic function in AD is worth exploring.

Broadly speaking, the majority of work to examine the pathologies underpinning AD have tended to focus upon amylogenic pathways. The majority of these model the amyloid aspects of AD through Aβ plaques. Despite many transgenic mouse models of AD existing, previous generations of Aβ mice have achieved their Aβ overexpression by also overexpressing amyloid precursor protein (APP). Over time, it has become clear that APP overexpression alone can introduce confounds that make it difficult to dissociate the causal effects of Aβ compared to APP. Mice overexpressing human wild-type APP have cognitive impairments in the Morris water maze and object-recognition test, increased tau hyperphosphorylation and reduced GluA1, GluA2, GluN1, GluN2A, GluN2B, phosphorylated CaMKII and PSD95 (Simón et al., 2009). This was also associated with reduced cell density in the pyramidal layer of CA1 (Simón et al., 2009). Others have found that other Aβ mice (APP23 mice) produced higher amounts of APP fragments, including C-terminal fragment β/α and APP intracellular domain (Saito et al., 2014). The phenotypes associated with APP overexpression alone encapsulate what are known to be AD-specific pathologies, underlining the potential risks of using APP overexpression models to make AD interpretations.

Recently, a new generation of AD mouse models have become available that achieve Aβ pathologies without the overexpression of APP (Saito et al., 2014). This has been achieved by humanising the murine Aβ sequence through a KI strategy. Up to three familial mutations have been knocked-in to generate two AD mouse models. The *App^NL-G-F^* mouse contains the Swedish KM670/671NL mutation and the Iberian I716F mutation, which results in elevated total Aβ and elevated Aβ_42_/Aβ_40_ ratio, and the additional Artic mutation E693G, which promotes aggressive Aβ oligomerisation and amyloidosis (Saito et al., 2014). In *App^NL-G-F^* mice, Aβ plaques are saturated by 9 months of age. Hence, *App^NL-G-F^* mice are useful to understand the effects of plaque-based Aβ in the generation of AD-related pathologies.

Within the current study, we have sought to explore whether these new *App* KI mouse models display alterations in behaviours related to social motivation, social memory and anxiety. Briefly, in terms of behaviour, we found marked, yet seemingly contradictory, changes in anxiety tasks [‘anxiogenic’ in the open field (OF), ‘anxiolytic’ in the elevated plus maze (EPM)], together with mild deficits on social tasks, in *App^NL-G-F^* KI mice. Arguably, the marked anxiety-related behavioural changes pointed more towards changes in decision-making than towards a simple shift in core anxiety-related emotionality. Accordingly, we applied three methodological assays to these mice, probing aspects of anatomy, physiology and genetics, with each assay consistently targeting both the prefrontal cortex (which has long been linked to decision-making interfacing affect and cognition) and the basolateral amygdala (which has long been linked to anxiety-related emotionality). Consistent with decision-making being affected more than core emotionality (albeit with other possible interpretations), we found that the prefrontal cortex exhibited alterations in all three of our assays (microstructure, glutamatergic-dependent gamma oscillations and glutamatergic gene expression), while the basolateral amygdala exhibited alterations in none of them. We conclude that insoluble Aβ overexpression leads to alterations in prefrontal cortices (as well as the hippocampus), which could at least partially underlie changes in social and anxiety-related tasks.

## RESULTS

### *App^NL^^-G-F^* KI mice show changes in anxiety-assessing tasks

**Fig. 1.**
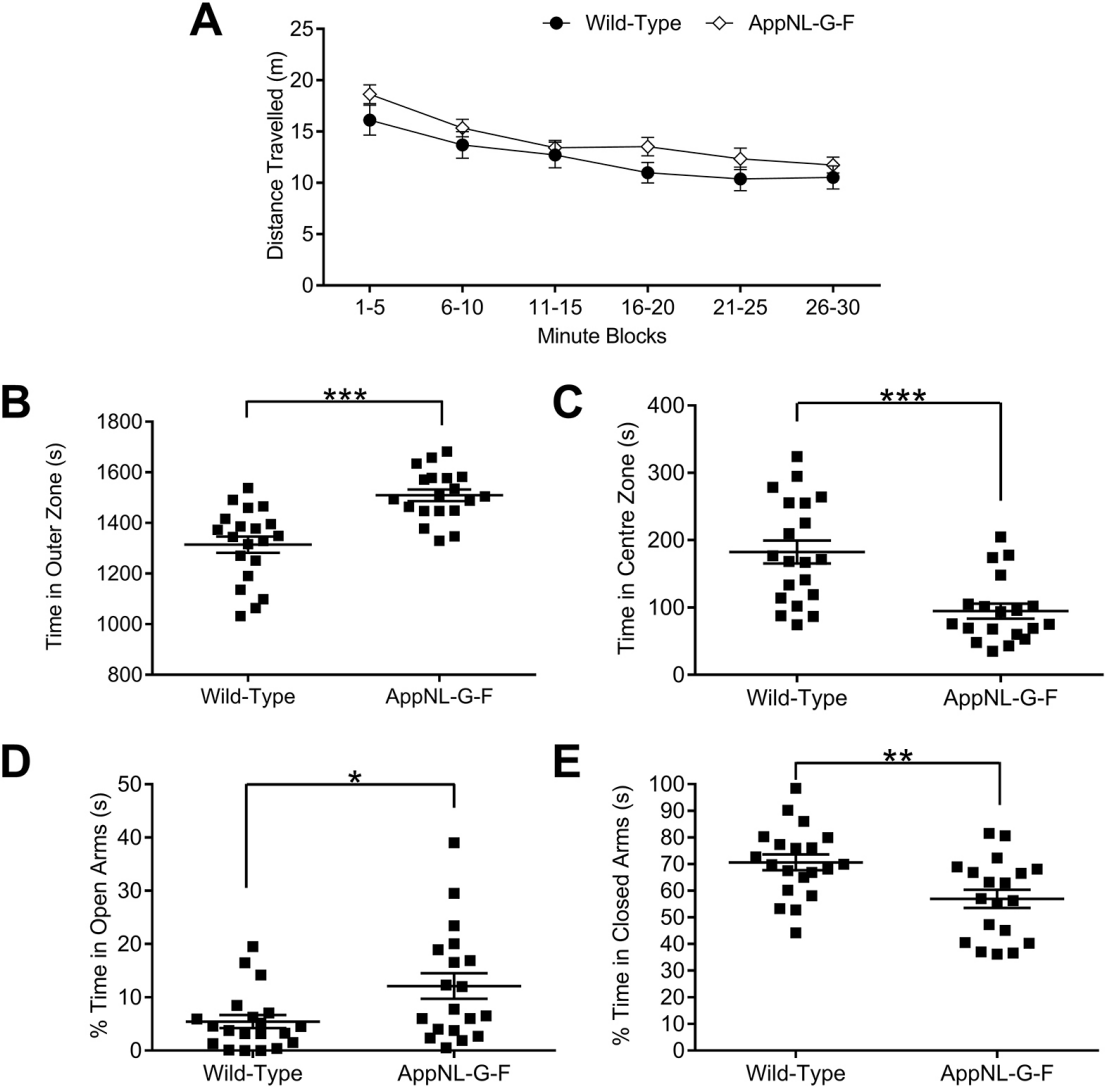
**Anxiety behaviours in 8-month-old *App^NL-G-F^*** **KI mice in the open field (OF) and elevated plus maze (EPM).** (A) Given 30 min free ambulation, all genotypes expressed similar amounts of locomotor activity as quantified by distance travelled. (B,C) *App^NL-G-F^* KI mice displayed thigmotaxis in the OF, spending significantly more time against the walls (B) and less time in the centre zone (C). (D,E) Conversely, *App^NL-G-F^* KI mice spent significantly more time exploring the open arms of the EPM (D) and significantly less time within the closed arms (E), suggesting an anxiolytic profile. All statistics used two-way ANOVAs. ***P<0.001, **P<0.01, *P<0.05. Error bars are s.e.m. Wild-type *n*=21, *App^NL-G-F^* KI *n*=19.

To assess the potential role of Aβ in anxiety, we undertook two behavioural paradigms that are widely used to probe anxiety in rodents: the OF test and the EPM. In the OF, mice were allowed to freely ambulate for 30 mins, during which we measured the distance they travelled in 5 min blocks. We observed that *App^NL-G-F^* KI mice travelled a similar distance to wild-type mice (Fig. 1A; genotype *F*_(1,35)_=1.93, *P*=0.174, time block × genotype × sex *F*_(1,55)_ <1, *P*=0.703, genotype × sex *F*_(1,35)_=2.09, *P*=0.157), suggesting that *App*^*NL-G-F*^ KI mice do not have any overt deficiencies in motor function. We subsequently divided the floor of the arena into an outer zone, an intermediate zone and a centre zone (see Materials and Methods) to determine whether *App*^*NL-G-F*^ KI mice displayed a propensity to stay close to the walls (thigmotaxis) and avoid the centre. Notably, *App^NL-G-F^* KI mice spend markedly more time in the outer zone (i.e. against the walls) (Fig. 1B; genotype *F*_(1,35)_=24.47, *P*<0.0001, genotype × sex *F*_(1,35)_<1, *P*=0.994). Further suggesting an anxiogenic phenotype, *App^NL-G-F^* KI mice spent significantly less time in the centre zone (Fig. 1C; genotype *F*_(1,35)_=18.36, *P*<0.0001, genotype × sex *F*_(1,35)_<1, *P*=0.832). Although no differences between the genotypes were observed for entries to the outer zone, *App^NL-G-F^* KI mice made fewer entries to the centre zone (Fig. S1A,B, see figure legend for statistics). Based on both outer zone entries and total distance travelled (see above), it is unlikely that hypo- or hyperactivity in *App^NL-G-F^* KI mice can explain the observed behavioural differences.

We next examined whether *App^NL-G-F^* KI mice also had a similarly anxiogenic profile in the EPM. Surprisingly, *App^NL-G-F^* KI mice spent significantly more time in the EPM's open arms (Fig. 1D; genotype *F*_(1,35)_=6.51, *P*=0.015, genotype × sex *F*_(1,35)_<1, *P*=0.578) and markedly less time in the EPM's closed arms (Fig. 1E; genotype *F*_(1,35)_=9.05, *P*=0.005, genotype × sex *F*_(1,35)_<1, *P*=0.651). Furthermore, *App^NL-G-F^* KI mice spent markedly less time in the centre zone (see Fig. S1C for statistics), and made significantly more head dips (see Fig. S1D for statistics). As with the OF, the specificity of the differences is unlikely to be explained by hyper- or hypoactivity, as *App^NL-G-F^* KI mice travelled a similar distance within the test (see Fig. S1E for statistics). In summary, *App^NL-G-F^* KI mice show anxiety-related behaviours that differ between experimental paradigms; anxiogenic in the OF, anxiolytic in the EPM.

**Fig. 2.**
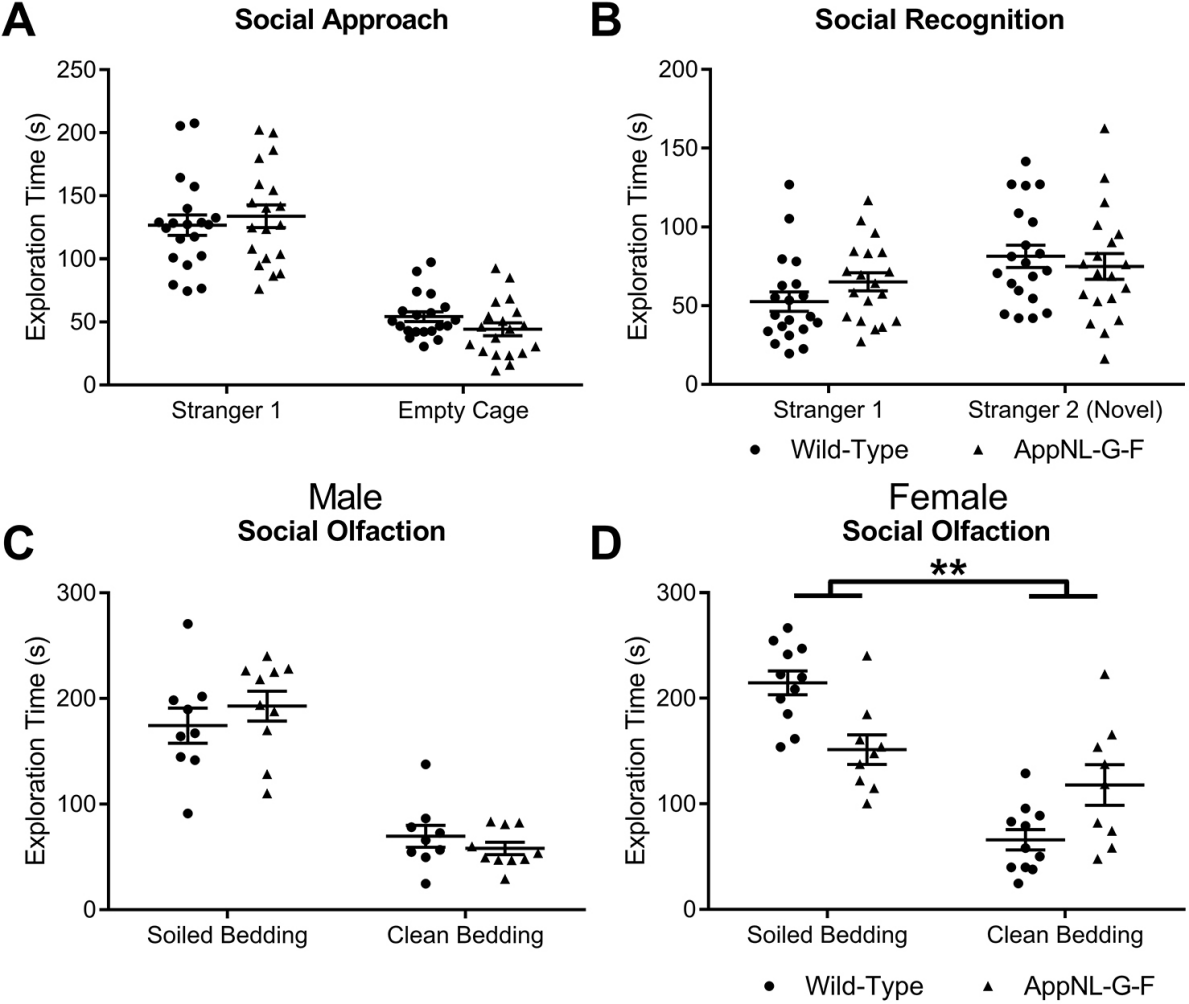
**Social behaviours in *App^NL-G-F^*** **KI mice.** (A) All genotypes showed a similar preference for exploring a novel, same-sexed novel conspecific (Stranger 1), compared to an empty cage. (B) However, *App^NL-G-F^* KI mice displayed a marginally weaker discrimination between the previously explored mouse (Stranger 1) and a second novel conspecific (Stranger 2). (C,D) Mice were then required to discriminate between a social smell (soiled bedding) or a non-social smell (clean bedding). Although all male genotypes show a similar preference for exploring the social cue compared to male wild-type mice (C), female *App^NL-G-F^* KI mice show significantly weaker discrimination compared to female wild-type mice (D). All statistics used two-way ANOVAs. **P<0.01. Error bars are s.e.m. Wild-type: male *n*=9, female *n*=11; *App^NL-G-F^* KI: male *n*=10, female *n*=9.

To explore whether social motivation was altered in *App* KI mice, we examined sociability in the three-chambered social approach test (Moy et al., 2004; Nadler et al., 2004), which exploits the preference of a mouse to explore a novel mouse enclosed within a wire cage compared to an identical empty cage. All genotypes showed a clear, similar preference for exploring the cage containing the novel mouse (Fig. 2A: genotype *F*_(1,35)_<1, *P*=0.670, discrimination between the novel mouse and empty cage *F*_(1,35)_=123.88, *P*<0.0001, genotype × discrimination between the novel mouse and empty cage *F*_(1,35)_=1.33, *P*=0.257, genotype × sex *F*_(1,35)_=1.36, *P*=0.252).

Next, we tested whether *App* KI mice were able to show social novelty recognition by preferentially exploring a second novel conspecific over a previously explored conspecific (Fig. 2B). We found that discrimination was, albeit not significantly, weaker in *App^NL-G-F^* KI mice (*F*_(1,35)_=3.42, *P*=0.073). There were no significant differences between genotype and sex (*F*_(2,35)_<1, *P*=0.955), nor was there any difference in genotypic discrimination of the novel and familiar mouse by sex (genotype × discrimination between the novel mouse and familiar mouse × sex *F*_(1,35)_<1, *P*=0.681). Thus, only subtle deficits exist for social recognition memory in *App^NL-G-F^* KI mice.

Finally, we tested whether *App* KI mice showed motivation to explore a social smell (soiled bedding) compared to a non-social smell (clean bedding). Discrimination between the social and non-social olfactory stimulus differed by sex and genotype (genotype × discrimination × sex *F*_(1,35)_=12.50, *P*=0.001, genotype × discrimination *F*_(1, 35)_=4.30, *P*=0.045). To investigate the source of difference, we performed further statistical analysis on whether social olfaction performance of *App^NL-G-F^* KI mice differed by sex. For males (Fig. 2C), both genotypes showed discrimination for the social olfactory cue (simple effects analysis, *F*_(1,35)_<1). However, female *App^NL-G-F^* KI mice displayed significantly weaker discrimination between the social and non-social olfactory stimulus compared to female wild-type mice (Fig. 2D; simple effects analysis, *F*_(1,35)_=10.55, *P*=0.003).

### *App^NL-G-F^* KI mice have microstructural changes in the prefrontal cortex and hippocampus

Given the behavioural changes in the OF, EPM and social olfaction test, we decided to explore putative neurobiological mechanisms that may explain these impairments. Our approach was to examine the integrity of brain regions associated with both social and anxiety behaviours (see Materials and Methods). These centred upon the prefrontal cortex [orbitofrontal cortex (OFC) and ACC], the hippocampus (anterior and posterior) and the amygdala [including the basolateral amygdala (BLA)]. To derive quantitative measures of diffusion tensor imaging (DTI), we examined fractional anisotropy (FA) and mean diffusivity (MD), examining diffusion across the λ_1_, λ_2_ and λ_3_ vectors, followed by axial diffusivity (AxD) and radial diffusivity (RD) to determine preferential diffusion along the λ_1,_ or λ_2_ and λ_3_ vectors, respectively.

Given that the amygdala has been widely associated with both social and anxiety behaviours (Adolphs, 2010; Davis, 1992; Davis et al., 1994), we segmented the whole amygdala region, in anterior and posterior planes, and, separately, the BLA, to determine whether structural alterations may explain the behavioural changes. For all measures (FA, MD, AxD and RD), in both the anterior and posterior amygdala, plus the BLA, we did not observe any significant changes in tissue diffusion properties (Table S1 for non-significant statistics and Fig. S2).

**Fig. 3.**
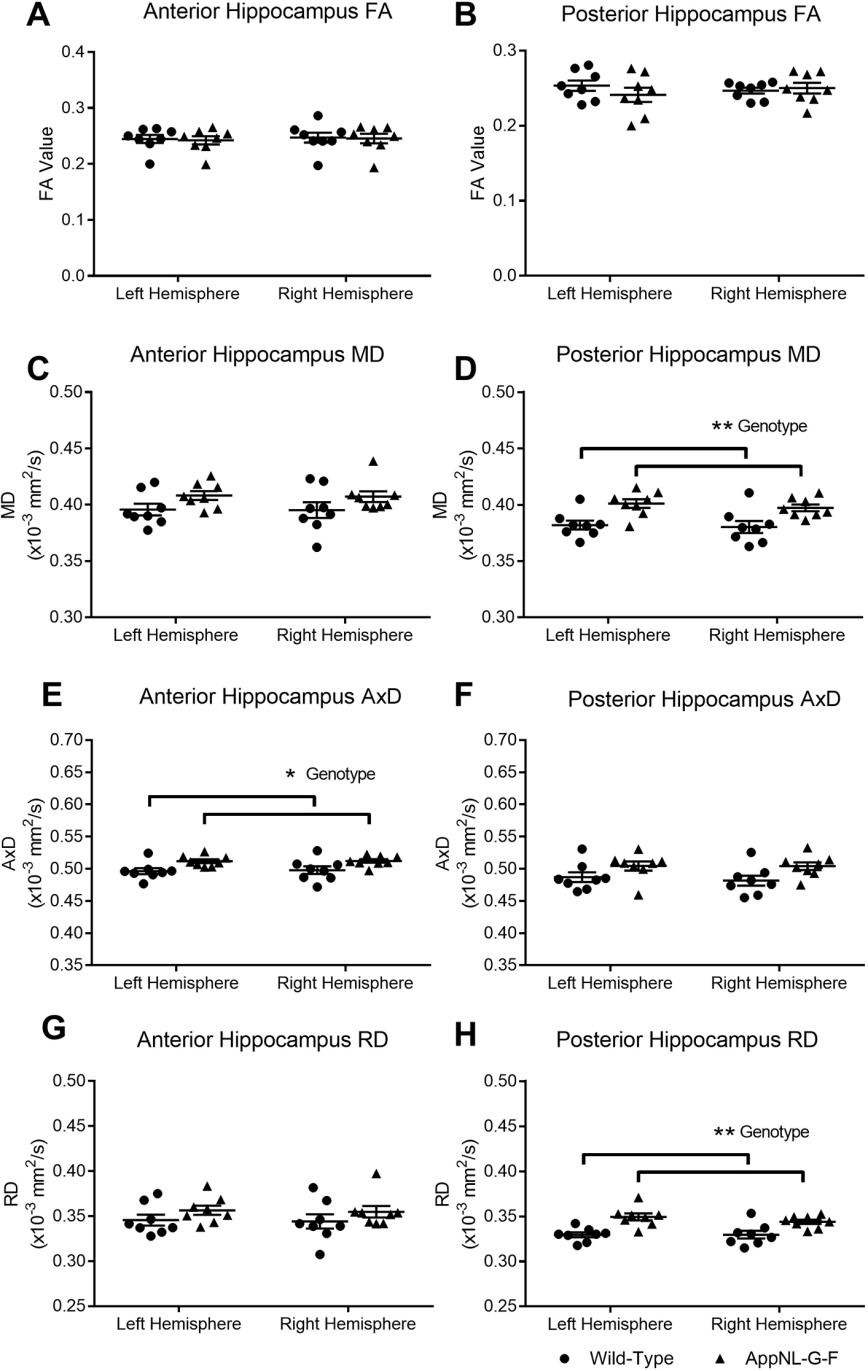
**Alterations in fractional anisotropy (FA) and mean diffusivity (MD) in the hippocampus.** Diffusion tensor imaging (DTI) images of the hippocampus were segmented at two regions: anterior (bregma −1.94 mm) and posterior (bregma −3.28 mm). FA was not significantly altered between the wild-type and *App^NL-G-F^* KI mice in the anterior (A) and posterior (B) hippocampus. MD of the anterior hippocampus did not differ between the genotypes (C); however, MD in the posterior hippocampus was significantly higher in *App^NL-G-F^* KI mice (D). Diffusivity was further characterised by examining axial (AxD) and radial (RD) diffusivity. AxD was significantly increased in the anterior hippocampus of *App^NL-G-F^* KI mice (E), but not in the posterior hippocampus (F). RD did not differ between the genotypes in the anterior hippocampus, but *App^NL-G-F^* KI mice had significantly increased RD in the posterior hippocampus compared to wild-type mice. All statistics used two-way ANOVAs. **P<0.01, *P<0.05. Error bars are s.e.m. Wild-type *n*=8, *App^NL-G-F^* KI *n*=8.

Ventral hippocampal regions of the posterior hippocampus are associated with unconditioned anxiety as tested by OF and EPM tasks (Bannerman et al., 2004), whilst dorsal and ventral regions are increasingly being associated with social recognition across the whole hippocampal extent (Hitti and Siegelbaum, 2014; Okuyama et al., 2016). We therefore next examined the microstructure of the hippocampus along the anterior to posterior axis. FA of the anterior (Fig. 3A) and posterior (Fig. 3B) hippocampus did not differ between wild-type and *App*^*NL-G-F*^ KI mice (Table S2 for non-significant statistics). However, whilst MD in the anterior hippocampus (Fig. 3C) was similar between the genotypes (*F*_(1,14)_<1, *P*=0.263), MD was significantly higher in the posterior hippocampus (Fig. 3D) of *App^NL-G-F^* KI mice (*F*_(1,14)_=12.18, *P*=0.011). For AxD, *App^NL-G-F^* KI mice had significantly increased diffusion in the anterior hippocampus (Fig. 3E; *F*_(1,14)_=7.55, *P*=0.047) but not in the posterior hippocampus (Fig. 3F; *F*_(1,14)_=4.45, *P*=0.068). RD was significantly higher in *App^NL-G-F^* KI mice in the posterior hippocampus (Fig. 3G; *F*_(1,14)_=18.13, *P*=0.005) but not the anterior hippocampus (Fig. 3H; *F*_(1,14)_=1.52, *P*=0.142). In summary, diffusion changes in the hippocampus were not marked, with significant changes only in anterior hippocampal AxD and posterior hippocampal RD.

**Fig. 4.**
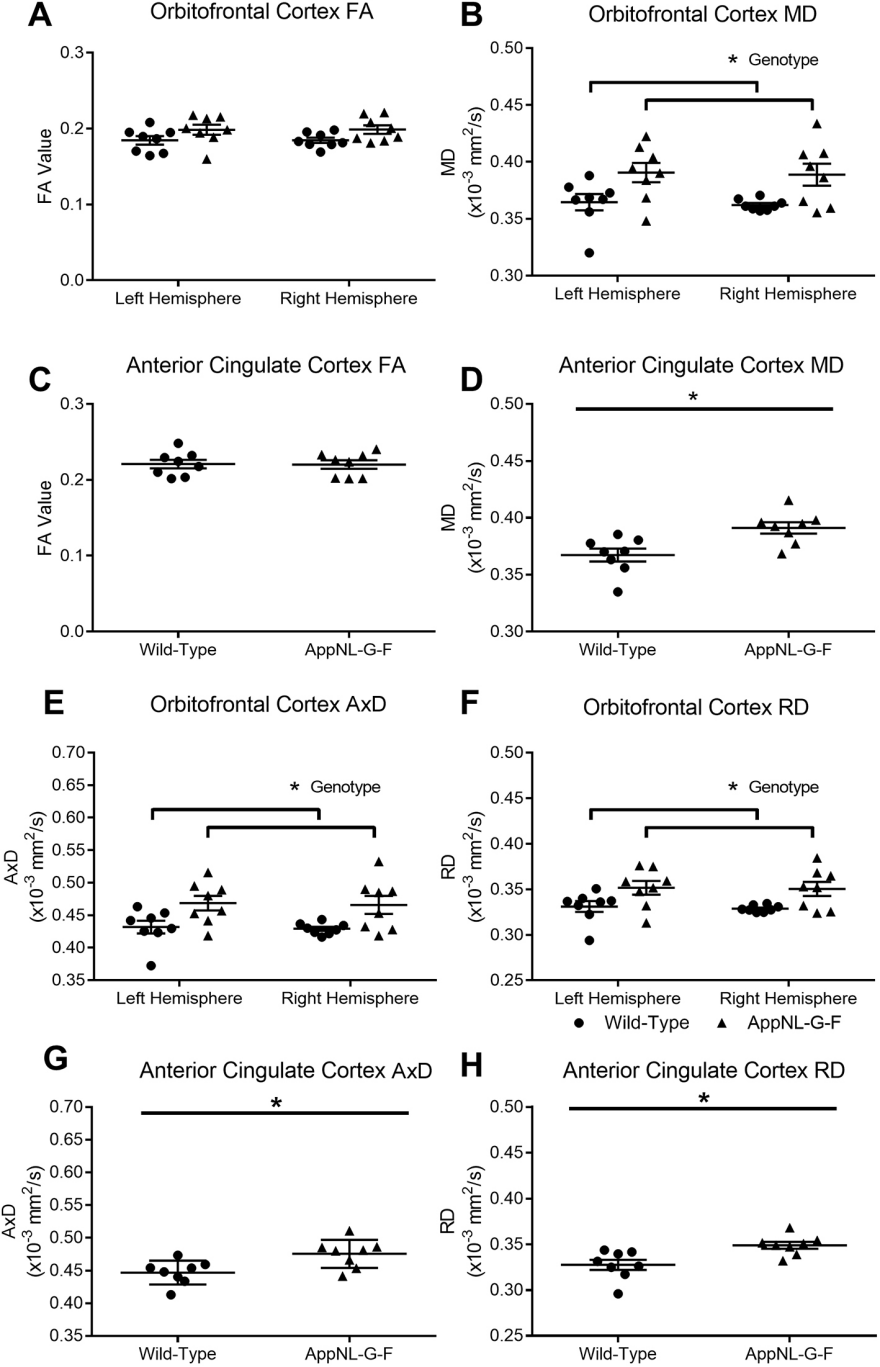
**Alterations in fractional anisotropy (FA) and mean diffusivity (MD) in the orbitofrontal cortex and anterior cingulate cortex (ACC) of the prefrontal cortex.** Diffusion tensor imaging (DTI) images were segmented for the orbitofrontal cortex at bregma +2.58 mm and for the ACC at bregma +1.18 mm. FA was not significantly altered between wild-type and *App^NL-G-F^* KI mice in the orbitofrontal cortex (A); however, MD was significantly higher for *App^NL-G-F^* KI mice (B). Similarly, FA did not differ between the genotypes for the ACC (C), but MD was significantly higher in *App^NL-G-F^* KI mice (D). Axial (AxD) and radial (RD) diffusivity was then analysed. In *App^NL-G-F^* KI mice, AxD (E) and RD (F) were both significantly increased in the orbitofrontal cortex. AxD (G) and RD (H) were also both increased in *App^NL-G-F^* KI mice in the ACC. A, B, E and F used two-way ANOVAs. C, D, G and H used *t*-tests. *P<0.05. Error bars are s.e.m. Wild-type *n*=8, *App^NL-G-F^* KI *n*=8.

We next segmented two prefrontal cortical regions: the OFC and the ACC. In addition to roles for OFC and ACC regions in anxiety and social behaviours, resting-state functional magnetic resonance imaging (rsfMRI) has shown that connectivity of the medial prefrontal cortex to other regions is abnormal in *App^NL-G-F^* KI mice, with the ACC being the most altered region (Latif-Hernandez et al., 2017). However, it is currently unknown as to whether structural changes in the ACC contributed to the rsfMRI result. In the OFC of *App^NL-G-F^* KI mice, there were no significant differences in FA (Fig. 4A; genotype *F*_(1,14)_=4.51, *P*=0.063); however, MD was significantly increased (Fig. 4B; genotype *F*_(1,14)_=8.61, *P*=0.026). Similarly, for the ACC, FA was similar between the genotypes (Fig. 4C; genotype *t*_(14)_ <1, *P*=0.37) but MD was significantly increased (Fig. 4D; genotype *t*_(14)_=3.13, *P*=0.021). We further explored prefrontal cortical changes by quantifying AxD and RD. In the OFC, both AxD and RD were significantly increased in *App^NL-G-F^* KI mice [*F*_(1,14)_=8.34, *P*=0.032 (Fig. 4E) and *F*_(1,14)_=8.23, *P*=0.042 (Fig. 4F), respectively]. AxD and RD were also significantly increased in the ACC of *App^NL-G-F^* KI mice [*t*_(14)_=2.88, *P*=0.037 (Fig. 4G) and *t*_(14)_=3.19, *P*=0.016 (Fig. 4H)]. In summary, for both the OFC and ACC, FA was not significantly altered, but MD, AxD and RD were all increased in *App^NL-G-F^* KI mice. Significant increases in three of these DTI measures likely reflects widespread pathology in the OFC and ACC of *App^NL-G-F^* KI mice. The DTI-related alterations in these prefrontal regions notably contrasted with an absence of any alterations in DTI measures in the amygdala.

Finally, we performed amyloid plaque staining on the corresponding tissue sections that were analysed for DTI. By 9 months of age in *App^NL-G-F^* KI mice, the OFC, ACC, amygdala and hippocampus all exhibit substantial plaque load (Fig. S3), indicating that the lack of DTI changes in the amygdala are not due to an absence of amyloid plaques.

### Alterations in prefrontal cortical NMDA-dependent gamma oscillations in *App^NL-G-F^* KI mice

Although some microstructural changes were detected in the hippocampus of *App^NL-G-F^* KI mice (anterior hippocampal AxD and posterior hippocampal RD), these were relatively inconsistent compared to the alterations observed within the prefrontal cortex. Another study found the ACC as being the most significantly altered brain region in *App^NL-G-F^* KI mice as detected by rsfMRI (Latif-Hernandez et al., 2017). This suggests that substantial changes are occurring in the ACC in *App^NL-G-F^* KI mice, as broadly consistent with the ACC Aβ load being high in MCI, and predicting faster conversion to AD (Okello et al., 2009). As such, and given the importance of the ACC and amygdala to anxiety and social behaviours, we decided to contrast these two regions to examine whether altered network physiology might accompany DTI-derived microstructural alterations.

**Fig. 5.**
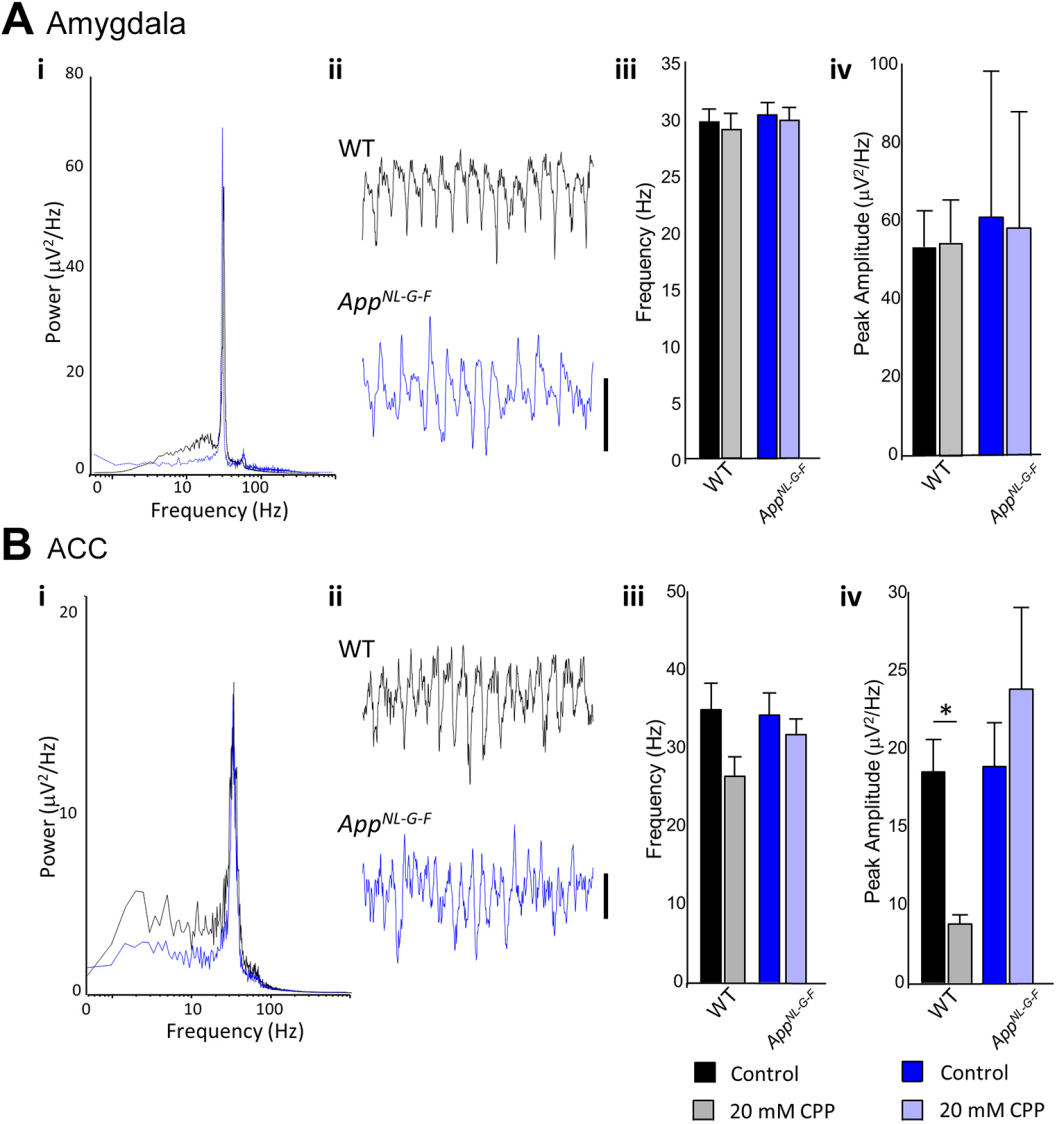
**Gamma oscillations in the amygdala and anterior cingulate cortex (ACC).** (Ai) Pooled power spectrum of gamma oscillatory activity in the basolateral amygdala in wild-type (WT; black) and *App^NL-G-F^* KI (blue) mice. (Aii) Example traces showing 500 ms of gamma oscillatory activity in WT (black) and *App^NL-G-F^* KI (blue) mice. Scale bar: 50 mV. (Aiii) Graph showing the effect of NMDA receptor antagonism (20 mM CPP) on the frequency of basolateral amygdala gamma oscillations (see key at bottom of figure). (Aiv) Graph showing the effect of NMDA receptor antagonism (20 mM CPP) on the amplitude of basolateral amygdala gamma oscillations. (Bi) Pooled power spectrum of gamma oscillatory activity in the ACC in WT (black) and *App^NL-G-F^* KI (blue) mice. (Bii) Example traces showing 500 ms of gamma oscillatory activity in WT (black) and *App^NL-G-F^* KI (blue) mice. Scale bar: 20 mV. (Biii) Graph showing the effect of NMDA receptor antagonism (20 mM CPP) on the frequency of ACC gamma oscillations. (Biv) Graph showing the effect of NMDA receptor antagonism (20 mM CPP) on the amplitude of ACC gamma oscillations. Key: dark colours represent control recordings, light colours represent recordings with CPP. All statistics used *t*-tests. *P<0.05. Error bars are s.e.m. Wild-type *n*=5, *App^NL-G-F^* KI *n*=5.

We generated gamma oscillations in brain slices using kainate and tested their dependency on NMDA receptors, which have previously been shown to modulate peak frequency through inhibitory postsynaptic currents (Carlén et al., 2012; McNally et al., 2011), by modifying recruitment of different interneuron subpopulations (Middleton et al., 2008). Gamma oscillations in non-pharmacologically treated slices were generally, in terms of peak amplitude and frequency, very similar between wild-type and *App^NL-G-F^* KI mice in both the BLA and the ACC. Within the BLA of the amygdala, we found that, in wild-type mice, peak amplitude and frequency of gamma oscillations were unaffected by the application of the broad-spectrum NMDA receptor antagonist CPP (Fig. 5Aiii; *t*_(14)_<1, *P*=0.991 and *t*_(14)_<1, *P*=0.656, respectively). We observed similar results in the *App^NL-G-F^* KI mice, with peak amplitude and frequency unaffected by NMDA antagonism (Fig. 5Aiv; *t*_(14)_<1, *P*=0.951 and *t*_(14)_<1, *P*=0.709, respectively). Together, this suggests that gamma oscillations in the BLA in *App^NL-G-F^* KI mice are unaffected by Aβ deposition.

Next, we studied gamma oscillations within the ACC of the prefrontal cortex. In wild-type mice, the frequency of gamma oscillations was unaffected by CPP (Fig. 5Biii; *t*_(14)_=1.91, *P*=0.086). However, gamma peak amplitude was significantly reduced by CPP application (Fig. 5Biv; *t*_(14)_=2.96, *P*=0.014), suggesting that, as expected (e.g. Carlén et al., 2012), ACC gamma oscillations normally require NMDA receptors in wild-type conditions. In contrast, in *App^NL-G-F^* KI mice, gamma amplitude (and frequency) were unaltered by NMDA receptor antagonism (*t*_(14)_<1, *P*=0.404; *t*_(14)_<1, *P*=0.430, respectively), suggesting that ACC gamma oscillations in *App^NL-G-F^* KI mice have lost their dependency upon NMDA receptors. In summary, the contribution of NMDA receptors to gamma oscillatory mechanisms appeared abnormally reduced in the prefrontal cortex.

### Prefrontal NMDA receptor expression is reduced in *App^NL-G-F^* KI mice

**Fig. 6.**
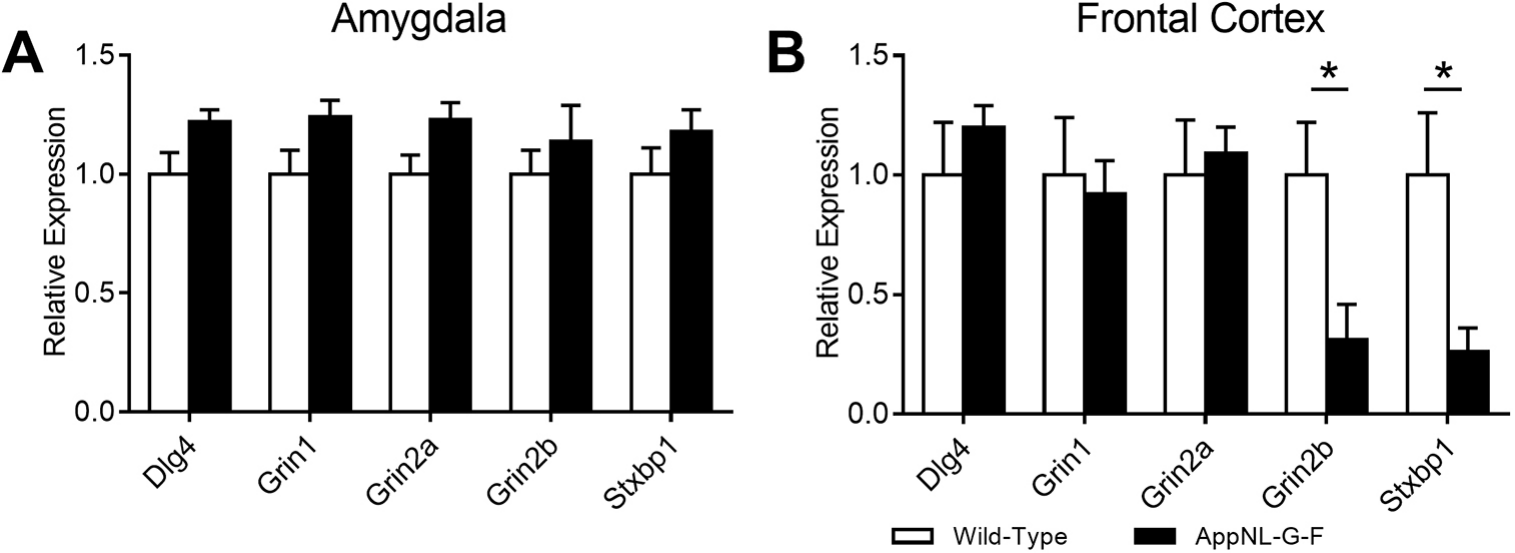
**Gene expression within the frontal cortex and amygdala.** (A) Although *App^NL-G-F^* KI mice (black bars) generally had higher expression of our selected genes within the amygdala, these were not significantly different to wild-type mice (white bars). (B) Within the frontal cortex, expression of both *Grin2b* and *Stxbp1* (Munc18-1) were significantly reduced in *App^NL-G-F^* KI mice. All statistics used *t*-tests. *P<0.05. Error bars are s.e.m. Wild-type *n*=6, *App^NL-G-F^* KI *n*=6.

To further understand this pattern of NMDA-receptor-related alterations in gamma oscillations in the prefrontal cortex but not the BLA, we next analysed the mRNA expression of synaptic genes, including NMDA receptors and pre- and postsynaptic receptors relating to NMDA receptor function. Within the amygdala, although we generally observed higher gene expression in *App^NL-G-F^* KI mice (Fig. 6A), no significant genotypic differences were observed for *Dlg4* (*t*_(10)_=2.14, *P*=0.058), *Grin1* (*t*_(10)_=1.97, *P*=0.078), *Grin2a* (*t*_(10)_=2.16, *P*=0.056), *Grin2b* (*t*_(10)_<1, *P*=0.455) or *Stxbp1* (*t*_(10)_=1.27, *P*=0.234). We next examined the mRNA expression in the frontal cortex (Fig. 6B). We did not observe any significant differences between wild-type and *App^NL-G-F^* KI mice for the genes *Dlg4* (*t*_(10)_<1, *P*=0.420), *Grin1* (*t*_(10)_<1, *P*=0.780) and *Grin2a* (*t*_(10)_<1, *P*=0.731). However, the expression of *Grin2b* (*t*_(10)_=2.59, *P*=0.027), which encodes the 2B subunit of the NMDA receptor, and *Stxbp1* (*t*_(10)_=2.66, *P*=0.024), which encodes Munc18-1, was significantly reduced in *App^NL-G-F^* KI mice. Together, the change in the dependency of NMDA-receptor-mediated gamma rhythms in the ACC of *App^NL-G-F^* KI mice could be related to reduced *Grin2b* or presynaptic release through Munc18-1.

## DISCUSSION

Within the current study, we have further characterised the behavioural profile of a new generation of *App* KI mice. When tested at 8 months, *App^NL-G-F^* KI mice show changes in behaviour relative to controls. *App^NL-G-F^* KI mice exhibited deficits in social olfactory discrimination, but not in sociability. In anxiety-assessing tasks, *App^NL-G-F^* KI mice exhibited task-dependent changes: (1) in the OF, they showed increased thigmotaxis and reduced centre time, i.e. an ostensibly anxiogenic profile; (2) in the EPM, they showed reduced closed-arm, and increased open-arm, time, i.e. an ostensibly anxiolytic profile. We discuss this seemingly contradictory profile further below. Briefly, one view is that these anxiety-related changes in *App^NL-G-F^* KI mice, being contradictory, likely do not reflect a simple shift towards one or other extreme of emotionality. Rather, their combined ostensibly anxiogenic OF, yet ostensibly anxiolytic EPM, profile could hint at altered mechanisms affecting decision making (e.g. ‘disinhibition’ in the EPM). If so, this idea would predict mild/absent amygdalar changes combined with marked prefrontal cortical changes. Consistent with this idea, alterations in microstructure, glutamatergic-dependent gamma oscillations, and glutamatergic gene expression were all observed in the prefrontal cortex, but not the amygdala, of *App^NL-G-F^* KI mice. Specifically, in *App^NL-G-F^* mice: DTI detected increases in MD, RD and AxD in the OFC and ACC; slice recordings showed that ACC prefrontal gamma mechanisms were unusual in being NMDA-receptor independent; and, finally, likely relatedly, quantitative real-time PCR (qPCR) showed reduced expression of *Grin2b* (encoding the 2B subunit of the NMDA receptor) in the prefrontal cortex. We speculate that the NMDA-receptor-independent ACC gamma oscillations could reflect compensatory changes following reduced NMDA receptor expression.

Within the current study, we observed a conflicting phenotype between increased thigmotaxis in the OF (anxiogenic) and increased time in the open arms in the EPM (anxiolytic). Others have examined the anxiety profile in *App^NL-G-F^* KI mice using the OF and found that 6-month-old mice spent more time in the OF centre zone (Latif-Hernandez et al., 2017). However, others have found no difference in time spent in the centre of an OF for 6-month-old *App^NL-G-F^* KI mice (Whyte et al., 2018). Latif-Hernandez et al. (2017) and Whyte et al. (2018) did not specifically quantify thigmotaxis in the OF, limiting comparisons to our study. Interestingly, locomotion within the OF appears to vary by age. Distance travelled in the OF is significantly greater in 6-month-old *App^NL-G-F^* KI mice, whereas, at 8 months (the current study) and 10 months of age, there were no significant differences (Latif-Hernandez et al., 2017; Whyte et al., 2018), suggesting hyperactivity is only detectable in younger mice.

Like our study, Latif-Hernandez et al. (2017) also used the EPM to explore anxiety behaviours in *App^NL-G-F^* KI mice. However, unlike us, they compared the behaviour of *App^NL-G-F^* KI mice to that of *App^NL^* KI mice only, and not to control wild-type mice. They found that *App^NL-G-F^* KI mice spent more time in the open arms at 6 months of age (Latif-Hernandez et al., 2017), which we replicated at 8 months of age. A very recent study has also found that 6- to 9-month-old *App^NL-G-F^* KI mice spent more time in the EPM open arms (Sakakibara et al., 2018). Together with our study, then, three independent studies suggest that *App^NL-G-F^* KI mice display an anxiolytic profile in the EPM. The ostensible contradiction between our results for the *App^NL-G-F^* KI mice in the OF (higher thigmotaxis) and EPM (lower centre time, higher open-arm time) is curious but not unique. Tg2576 mice, an AD model overexpressing mutant APP with the Swedish KM670/671NL mutation, also show OF thigmotaxis yet increased time in the EPM open arms (Lalonde et al., 2003). It is possible that the increased time spent in the open arms reflects a disinhibition phenotype. Disinhibition is a well-established, albeit less common, AD phenotype (Chung and Cummings, 2000; Hart et al., 2003), which could be manifested within the current study as a failure to inhibit the choice to enter the open arm. Given the lack of specific data to speak to a disinhibition hypothesis, it is clear that future anxiety testing in *App^NL-G-F^* KI mice will require multiple paradigms to clearly delineate anxiety from disinhibition.

Could perseveration help to explain the seemingly contradictory profiles we observed in *App^NL-G-F^* KI mice, i.e. the ostensibly anxiogenic profile in the OF and the ostensibly anxiolytic profile in the EPM? Prefrontal cortex damage can lead to perseverative behaviour, and we show prefrontal cortex alterations in *App^NL-G-F^* KI mice. Several studies find that dopamine transporter knock-out (DAT-KO) mice, which have aberrant prefrontal connectivity (Zhang et al., 2010), exhibit perseverative and stereotyped behaviour (e.g. Fox et al., 2013; Pogorelov et al., 2005; Ralph et al., 2001). Perseverative behaviour sometimes co-occurs (Pogorelov et al., 2005), and sometimes does not co-occur (Fox et al., 2013; Powell et al., 2004), with thigmotaxis in the same OF test in these mice. However, such perseverative behaviour reliably co-occurs with hyperactivity and stereotyped movements; in contrast, we did not observe any such hyperactivity, and could see no sign whatsoever of any unusual patterns of locomotion in *App^NL-G-F^* KI mice. Moreover, most importantly, in a study that explicitly investigated anxiety-related profiles in DAT-KO mice (Pogorelov et al., 2005), the perseverative behaviour co-occurred with an anxiogenic profile in both the OF (more thigmotaxis) and the zero maze (reduced open-arm time), an equivalent of the EPM. Furthermore, diazepam normalised the DAT-KO versus wild-type differences in in the zero maze, which the authors interpreted as suggesting that there is a genuine anxiety-like characteristic in the DAT-KO mice (Pogorelov et al., 2005). Another mouse model incorporating prefrontal alterations and perseverative behaviour is the MAO-A^Neo^ mouse (Bortolato et al., 2011). This mouse showed somewhat higher thigmotaxis in the OF, but open-arm entries/time were perfectly normal in the EPM. In summary, then, the relationship between perseverative behaviour and anxiety measures is not straightforward, and there is no particular evidence to suggest that perseveration resolves the apparent ‘OF-anxiogenic versus EPM-anxiolytic’ discrepancy raised by our findings. To our knowledge, only amyloid-related AD mouse models show this contradictory profile.

Several other AD mouse models exhibit impairments in social behaviour, including APP_swe_/PS1 (Filali et al., 2011) and Tg2576 (Deacon et al., 2009) mice. Latif-Hernandez et al. (2017) examined social behaviours in *App^NL-G-F^* KI mice and found that, although not significant, the discrimination ratio for exploring a novel mouse compared to an empty cage, and for comparing between the previously explored mouse and a second novel mouse, had fallen to chance by 10 months of age. We found that *App^NL-G-F^* KI mice show robust preference for exploring a novel conspecific compared to an empty cage. We did, however, find that social preference, as measured by discriminating a social odour cue, was impaired in female *App^NL-G-F^* KI mice. A key difference between our study and Latif-Hernandez et al. (2017) is that they used only female mice. Our study, with mixed-sex groups, suggests that females show a greater impairment in social behaviour than males, which will be an important consideration for future studies. Generally, the use of single-sex groups has been a problem for other AD mouse model literature. To the best of our knowledge, only two studies have used mixed-sex groups to investigate social deficits. In both cases, aged 3xTg-AD mice (>12 months old) have shown female-specific differences in social engagement (Bories et al., 2012; Torres-Lista and Gimenez-Llort, 2019). Although our findings are corroborative with these studies, more work is required to understand the nature of socially relevant sex differences in AD.

The ACC and amygdala are brain regions that have been well established with mediating anxiety/fear and social behaviours (Davidson, 2002; Davis, 1992; Davis et al., 1994; Etkin et al., 2011). Additionally, the prefrontal cortex has a well-established link to behavioural decision making, especially for affective stimuli (Dias et al., 1996). This may relate to our proposed ‘disinhibition’ explanation for the dichotomy in our two anxiety tasks, highlighting the prefrontal cortex as the critical brain region in *App^NL-G-F^* KI mice. Furthermore, these areas are also susceptible to degeneration in early AD pathology (Huang et al., 2002; Poulin et al., 2011; Scheff and Price, 2001). With these findings in mind, we hypothesised that structural and functional alterations within the amygdala and medial prefrontal cortex could partly explain behavioural impairments. As it turned out, we found little evidence for neurodegeneration-linked changes in the amygdala, but did find such changes in the prefrontal cortex.

Our analysis of microstructural integrity using DTI revealed that the prefrontal cortex of *App^NL-G-F^* KI mice, including the OFC and ACC, was substantially altered, and the functional consequences of these changes to the ACC was that gamma oscillations lost their dependency upon NMDA receptors, notably GluN2B receptors. Thus far, there have been limited investigations into the biological pathways altered in *App^NL-G-F^* KI mice. Using rsfMRI, Latif-Hernandez et al. (2017) found that the cingulate cortex was the most significantly altered brain region, with no visible impairment in the amygdala. Although further work is required to definitively describe the physiological pathways that explain the altered behaviour in *App^NL-G-F^* KI mice, our findings present an important step in this process. Although other brain regions likely contribute to changes in anxiety and social behaviours [gamma oscillations have also been found altered in the medial entorhinal cortex of *App^NL-G-F^* KI mice (Nakazono et al., 2017)], the prefrontal cortex could represent a key region. Neural oscillations in the medial prefrontal cortex have clear links to anxiety behaviours, both in the OF and EPM (Adhikari et al., 2010), and medial prefrontal cortex gamma is required for the expression of social novelty (Cao et al., 2018). Additionally, the loss of GluN2B or its phosphorylation impairs social behaviour (Jacobs et al., 2015; Wang et al., 2011) and increases anxiety in the OF (Hanson et al., 2014) and the EPM (Delawary et al., 2010). Furthermore, NMDA receptors are necessary for gamma oscillations mediated by the goblet cell interneurons within the entorhinal cortex (Middleton et al., 2008), with specific antagonism of GluN2B significantly reducing hippocampal gamma power (Hanson et al., 2013). Together, insoluble Aβ overexpression in *App^NL-G-F^* KI mice results in prefrontal cortical alterations, and this may be in part be through gamma oscillatory impairments by reduced GluN2B expression. This indicates that social and anxiety behavioural impairments in *App^NL-G-F^* KI mice may be driven by regions that do not include the amygdala, although further work will be required to corroborate this, including the specific antagonism of GluN2B using slice electrophysiology.

An important limitation of the current study is that we have not examined anatomical or neurobiological mechanisms by sex. Female *App^NL-G-F^* KI mice showed weaker performance in social olfactory discrimination, suggestive of sex differences. Future studies should consider the contribution of sex differences to both anatomical and physiological outputs in *App^NL-G-F^* KI mice. Additionally, our behavioural and neurobiological data varies at the time point in which the experiments took place (∼8 months for behaviour, ∼9 months for DTI and ∼12 months for electrophysiology). Thus, we cannot rule out a worsening/degenerating phenotype partly explaining the electrophysiological data.

A second limitation is that we have not performed physiological (i.e. electrophysiological and molecular) analysis on the hippocampus. Although DTI found alterations in the hippocampal microstructure of *App^NL-G-F^* KI mice, our aim herein was to examine brain regions more closely associated with anxiety and social behaviours, for which the hippocampus has some, but conflicting, evidence. Thus far, limited electrophysiological studies of the hippocampus have taken place in *App^NL-G-F^* KI mice, although long-term potentiation has been found reduced (Moriguchi et al., 2018). Thus, moving forwards, it will be important to examine the hippocampus in addition to prefrontal regions to ascertain how various brain regions contribute to the altered behaviours.

The findings presented herein show a clear dichotomy of anxiety behaviours between two different paradigms (anxiolytic-like in the EPM versus anxiogenic-like in the OF). We have argued that this pattern may be linked to degeneration in neural regions related to decision making, notably the medial prefrontal cortex, rather than to emotionality per se. We further postulate that microstructural integrity, gamma oscillatory function and *Grin2b* expression within the prefrontal cortex may, in part, explain these behavioural changes. Our data continues to highlight the importance of using AD models that do not have the limitation of APP overexpression. Further studies will be required to continue to refine the mechanisms that explain anxiety impairments in *App^NL-G-F^* KI mice.

## MATERIALS AND METHODS

### Ethics

All procedures were approved by the Durham University and University of York Animal Ethical and Welfare Review Boards, and were performed under UK Home Office Project and Personal Licenses in accordance with the Animals (Scientific Procedures) Act 1986.

### Experimental animals

#### Mice

Full details of the animals can be found elsewhere (Saito et al., 2014). Upon arrival at Durham, mice were backcrossed once to the C57BL/6J line, which was the same background strain as the previous institution where they were bred. Homozygote *App^NL-G-F^* KI and wild-type littermate mice were bred in house by heterozygote pairings and were weaned at P21 and ear biopsied for genotyping. Mice were housed with between two and five littermates, with a 12:12 light/dark cycle commencing at 07:00. Enrichment was provided with Perspex domes and shreddable nesting material. Genotyping protocols can be found elsewhere (Saito et al., 2014). In brief, the following primers were used (5′-3′): WT: TGTAGATGAGAACTTAAC, E16WT: ATCTCGGAAGTGAAGATG, loxP: CGTATAATGTATGCTATACGAAG and E16Mut: ATCTCGGAAGTGAATCTA. PCR was run at 53°C for 30 cycles for 30 s.

### Experimental design

#### Behaviour

In total, 21 wild-type [nine male (8.19±0.12 months; mean±s.e.m.), 11 female (8.10±0.04 months)] and 19 *App^NL-G-F^* homozygotes [ten male (7.93±0.17 months), nine female (7.44±0.43 months)] were used in behavioural testing by random allocation. Mice were transferred to the testing room and allowed at least 30 min habituation prior to testing. All mice were transferred to the apparatus via cardboard tubes. Experiments took place in the light phase between 09:00 and 17:00. All apparatus was cleaned with alcohol wipes between subjects. A minimum of 2 days was provided between experiments.

Testing procedures can be found elsewhere (Dachtler et al., 2014). All trials were recorded by Any-maze (Stoelting, Dublin, Ireland) tracking software. Mice undertook the following tests in this order: OF, EPM, social approach, social recognition and social olfactory discrimination. In brief, mice were placed into a 44 cm^2^ white Perspex arena and allowed free ambulation for 30 min. The floor of the arena was divided by Any-maze into an outer zone of 8 cm and a centre zone of 17.5 cm^2^ (note: we have not reported on the intermediate zone). Time spent and entries into these zones, along with distance travelled, were measured. The EPM, social approach, social recognition and social olfaction discrimination were run as in Dachtler et al. (2014), except that novel conspecifics and soiled bedding was sex matched with adult mice to the test subject. For the social tests, following a 15 min habituation, the test mouse was allowed 10 min of free exploration. The inter-trial interval between social novelty and social recognition experiments was 5 min. At the conclusion of testing, mice were used for electrophysiology, killed by perfusion fixation with 4% paraformaldehyde in phosphate buffered saline (PBS) or killed by cervical dislocation for molecular biology.

#### Diffusion tensor MRI

Eight wild-type [four male (9.02±0.63 months), four female (9.43±0.30 months)] and eight *App^NL-G-F^* homozygotes [four male (8.88±0.22 months), four female (9.89±0.21 months)] were used in MRI. Image acquisition has been described elsewhere (Pervolaraki et al., 2017, 2019). MRI was performed on a vertical 9.4 Tesla spectrometer (Bruker AVANCE II NMR, Ettlingen, Germany). During imaging, the samples were placed in custom-built MR-compatible tubes containing Fomblin Y (Sigma-Aldrich, Poole, Dorset, UK). The following acquisition protocol was used: TE: 35 ms, TR: 700 ms, 1 signal average. The field of view was set at 168×128×96 mm, with a cubic resolution of 62.5 μm/pixel and a B value of 1625 s/mm with 30 directions.

The *ex vivo* mouse brain 3D diffusion-weighted images were reconstructed from the Bruker binary file using DSI Studio (http://dsi-studio.labsolver.org). Directionally encoded colour map (DEC) images were generated by combining the information from the primary eigenvectors, diffusion images and the FA. Regions were extracted by manually segmenting OFC, ACC, hippocampal and whole-amygdalar regions using a mouse brain atlas (Franklin and Paxinos, 2008). We chose to focus on the aforementioned brain regions as these have been linked to social behaviour (Pervolaraki et al., 2019). Additionally, the hippocampus (Bannerman et al., 2004; McHugh et al., 2004), amygdala (Davis, 1992; Davis et al., 1994) and prefrontal cortical regions (including the ACC) (Davidson, 2002; Etkin et al., 2011) have all been linked to anxiety. Extraction of FA, MD, AxD and RD was performed within selected segmented brain regions, with three 100 µm sections (one anterior and one posterior to the segmented section) extracted. A full atlas-based description of the segmentation can be found in Pervolaraki et al. (2019).

#### Electrophysiology

Anterior cingulate or basolateral amygdala coronal slices (450 µm thick) were prepared from age-matched, 12-month-old male wild-type (*n*=5) mice and *App^NL-G-F^* KI (*n*=5) mice. Animals were anaesthetised with inhaled isoflurane, immediately followed by an intramuscular injection of ketamine (100 mg/kg body weight) and xylazine (10 mg/kg body weight). Animals were perfused intracardially with 50-100 ml of modified artificial cerebrospinal fluid (ACSF), which was composed of the following (in mM): 252 sucrose, 3 KCl, 1.25 NaH_2_PO_4_, 24 NaHCO_3_, 2 MgSO_4_, 2 CaCl_2_ and 10 glucose. The brain was removed and submerged in cold (4-5°C) ACSF during dissection. Once dissected, slices were maintained at 32°C in a standard interface recording chamber containing oxygenated ACSF consisting of (in mM): 126 NaCl, 3 KCl, 1.25 NaH_2_PO_4_, 1 MgSO_4_, 1.2 CaCl_2_, 24 NaHCO_3_ and 10 glucose. Persistent, spontaneous gamma oscillations were generated through the bath application of 400 nM kainic acid (KA). Further perfusion, through bath application of 20 mM 3-(2-carboxypiperazin-4-yl)propyl-1-phosphonic acid (CPP), was conducted to antagonise NMDA receptors. All salts were obtained from BDH Chemicals (Poole, UK) or Sigma-Aldrich (Poole, UK), and KA and CPP were obtained from BioTechne (MN, USA).

Extracellular field recordings were obtained using borosilicate micropipettes (Harvard Apparatus) filled with ACSF and had resistances of 2-5 MΩ. Recordings were bandpass filtered at 0.1-300 Hz. Power spectra were derived from Fourier transform analysis of 60 s epochs of data, and results were presented as mean±s.e.m.

#### RNA extraction and qPCR

For molecular biology, mice underwent cervical dislocation, with the brain extracted and placed into a mouse brain blocker (David Kopf Instruments, Tujunga, CA, USA). The olfactory bulbs were removed, followed by a section of tissue from bregma 3.56 to 2.58 mm, and from bregma −0.82 to −2.80 mm. The prefrontal cortex tissue was snap frozen. The posterior section was then further dissected with a scalpel to remove the amygdalar region, which was then snap frozen and stored at −80°C until use. Six wild-type [four male, two female (10.26±0.41 months)] and six *App^NL-G-F^* homozygotes [three male, three female (9.49±0.13 months)] were used for qPCR. Brain tissue of extracted regions (amygdala and prefrontal cortex) were homogenised by TissueRuptor drill (Qiagen, Manchester, UK), with ∼90 mg used for RNA extraction. Instructions were followed as per the Bio-Rad Aurum Total RNA Fatty and Fibrous Tissue Kit (Bio-Rad, Hemel Hempstead, UK, cat. # 7326830) and our previously optimised protocol (Pervolaraki et al., 2018). RNA quantity and quality were confirmed by Nanodrop spectrophotometer. cDNA was generated by the iScript cDNA Synthesis Kit (Bio-Rad, UK), with 1 µg of RNA used per reaction.

Primers (Table 1) were designed using Primer3 after identifying the gene sequence on NCBI (https://www.ncbi.nlm.nih.gov/). Primers (Integrated DNA Technologies, Leuven, Belgium) were tested for specificity and run conditions optimised by PCR using whole-brain cDNA. Plates were run with 10 µl per reaction, with 1 µl of cDNA, Bio-Rad SYBR Green (cat. # 1725121) and 300 nM primers. Samples were run in triplicate using the protocol of 95°C for 3 min, followed by 95°C for 10 s, 60°C for 30 s repeated 35 times. Gene expression was imaged using a Bio-Rad CFX Connect and analysed using Bio-Rad Connect Manager, and quantified using the 2^ΔΔCt^ method against the housekeeping gene *Gapdh*, which did not differ between the genotypes*.*

**Table 1.**
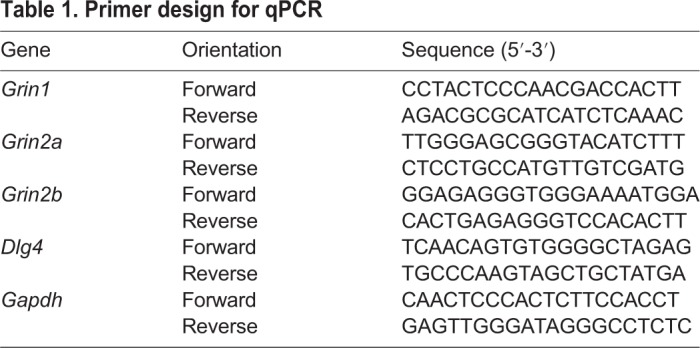
**Primer design for qPCR**

#### Immunostaining

The protocol for staining for amyloid plaques can be found elsewhere (Ly et al., 2011). Tissue from one wild-type and one *App^NL-G-F^* KI mouse previously scanned by MRI was washed and immersed in 30% sucrose for >48 h. Tissue was cryosectioned at 30 µm, followed by immersion in 88% formic acid for 15 min. Endogenous peroxidase activity was quenched with H_2_O_2_ for 30 min, followed by blocking in 2% bovine serum albumin (BSA) (Thermo Fisher Scientific) for 1 h at room temperature. Sections were then incubated with 1:500 6E10 (BioLegend, San Diego, CA, USA; cat. # 803001, lot # B225309) overnight at 4°C, followed by 1:500 biotinylated anti-mouse (Vector Labs, Peterborough, UK) for 2 h at room temperature. Sections were then reacted with Vectastain ABC kit (Vector Labs) followed by diaminobenzidine (DAB) treatment (Vector Labs) prior to mounting on slides for imaging by light microscopy.

### Data availability

Datasets will be made available subject to responsible request to J.D.

### Data analysis

All data are expressed as mean±s.e.m. Group sizes were calculated assuming an η^2^ of 0.14 for a predicted power of 0.8 with α=0.05. To assess the variance between genotypes within a single brain structure across hemispheres, data was analysed by within-subject repeated-measures two-way ANOVAs or unpaired *t*-tests. To correct for multiple comparisons, we employed the Benjamini-Hochberg procedure, with false discovery rate set to 0.4 (corrected *P*-values stated). Behaviour was analysed with ANOVAs, followed by tests of simple main effects. Non-significant statistical results, particularly hemisphere comparisons, can be found in the supplementary information. Statistical testing and graphs were made using GraphPad Prism version 6 and SPSS v22.

## Supplementary Material

Supplementary information
