# Supplementary material for: Insoluble Aβ overexpression in an App knock-in mouse model alters microstructure and gamma oscillations in the prefrontal cortex, affecting anxiety-related behaviours
Source: Dis Model Mech. 2019 Sep 24;12(9):dmm040550. doi: 10.1242/dmm.040550 (PMC6765200; doi:10.1242/dmm.040550)
Supplement: Supplementary information [file dmm-12-040550-s1.pdf]

Supplemental Material

Brain Region	DTI Measure	ANOVA Comparison	F Value	P Value
Anterior Amygdala	FA	Genotype	$F_{(1, 14)} < 1$	P = 0.384
		Hemisphere	$F_{(1, 14)} = 2.76$	P = 0.095
Posterior Amygdala	FA	Genotype	$F_{(1, 14)} < 1$	P = 0.300
		Hemisphere	$F_{(1, 14)} < 1$	P = 0.184
BLA	FA	Genotype	$F_{(1, 14)} < 1$	P = 0.268
		Hemisphere	$F_{(1, 14)} < 1$	P = 0.168
Anterior Amygdala	MD	Genotype	$F_{(1, 14)} < 1$	P = 0.179
		Hemisphere	$F_{(1, 14)} < 1$	P = 0.353
Posterior Amygdala	MD	Genotype	$F_{(1, 14)} < 1$	P = 0.274
		Hemisphere	$F_{(1, 14)} < 1$	P = 0.226
BLA	MD	Genotype	$F_{(1, 14)} = 3.23$	P = 0.084
		Hemisphere	$F_{(1, 14)} = 1.55$	P = 0.137
Anterior Amygdala	AD	Genotype	$F_{(1, 14)} < 1$	P = 0.173
		Hemisphere	$F_{(1, 14)} < 1$	P = 0.242
Posterior Amygdala	AD	Genotype	$F_{(1, 14)} < 1$	P = 0.221
		Hemisphere	$F_{(1, 14)} < 1$	P = 0.252
BLA	AD	Genotype	$F_{(1, 14)} = 5.81$	P = 0.053
		Hemisphere	$F_{(1, 14)} = 2.55$	P = 0.105
Anterior Amygdala	RD	Genotype	$F_{(1, 14)} < 1$	P = 0.189
		Hemisphere	$F_{(1, 14)} < 1$	P = 0.247
Posterior Amygdala	RD	Genotype	$F_{(1, 14)} < 1$	P = 0.332
		Hemisphere	$F_{(1, 14)} < 1$	P = 0.194
BLA	RD	Genotype	$F_{(1, 14)} = 2.67$	P = 0.100
		Hemisphere	$F_{(1, 14)} < 1$	P = 0.205

Table S1 Statistical analysis of the anterior (Bregma -1.94 mm) and posterior (Bregma -3.28 mm) amygdala, analysed for fractional anisotropy (FA), mean diffusion (MD), axial diffusion (AD) and radial diffusion (RD). Corrected P values stated (Benjamini-Hochberg corrected).

Disease Models & Mechanisms • Supplementary information

Brain Region	DTI Measure	ANOVA Comparison	F Value	P Value
Anterior Hippocampus	FA	Genotype	$F_{(1,14)}$ <1	P = 0.342
		Hemisphere	$F_{(1, 14)}$ = 2.18	P = 0.116
Posterior Hippocampus	FA	Genotype	$F_{(1, 14)}$ <1	P = 0.263
		Hemisphere	$F_{(1, 14)}$ <1	P = 0.305
Anterior Hippocampus	MD	Genotype	$F_{(1, 14)}$ = 2.89	P = 0.089
		Hemisphere	$F_{(1, 14)}$ <1	P = 0.311
Posterior Hippocampus	MD	Genotype	$F_{(1, 14)}$ = 12.18	P = 0.011
		Hemisphere	$F_{(1, 14)}$ = 1.07	P = 0.163
**Anterior Hippocampus**	**AD**	**Genotype**	**$F_{(1, 14)}$ = 7.56**	**P = 0.047**
		Hemisphere	$F_{(1, 14)}$ <1	P = 0.316
Posterior Hippocampus	AD	Genotype	$F_{(1, 14)}$ = 4.45	P = 0.068
		Hemisphere	$F_{(1, 14)}$ <1	P = 0.211
Anterior Hippocampus	RD	Genotype	$F_{(1, 14)}$ = 1.52	P = 0.142
		Hemisphere	$F_{(1, 14)}$ <1	P = 0.237
**Posterior Hippocampus**	**RD**	**Genotype**	**$F_{(1, 14)}$ <1**	**P = 0.005**
		Hemisphere	$F_{(1, 14)}$ <1	P = 0.158

Table S2 Statistical analysis of the anterior (Bregma -1.94 mm) and posterior (Bregma -3.28 mm) hippocampus for fractional anisotropy (FA), mean diffusion (MD), axial diffusion (AD) and radial diffusion (RD). Corrected P values stated (Benjamini-Hochberg corrected).

Disease Models & Mechanisms • Supplementary information

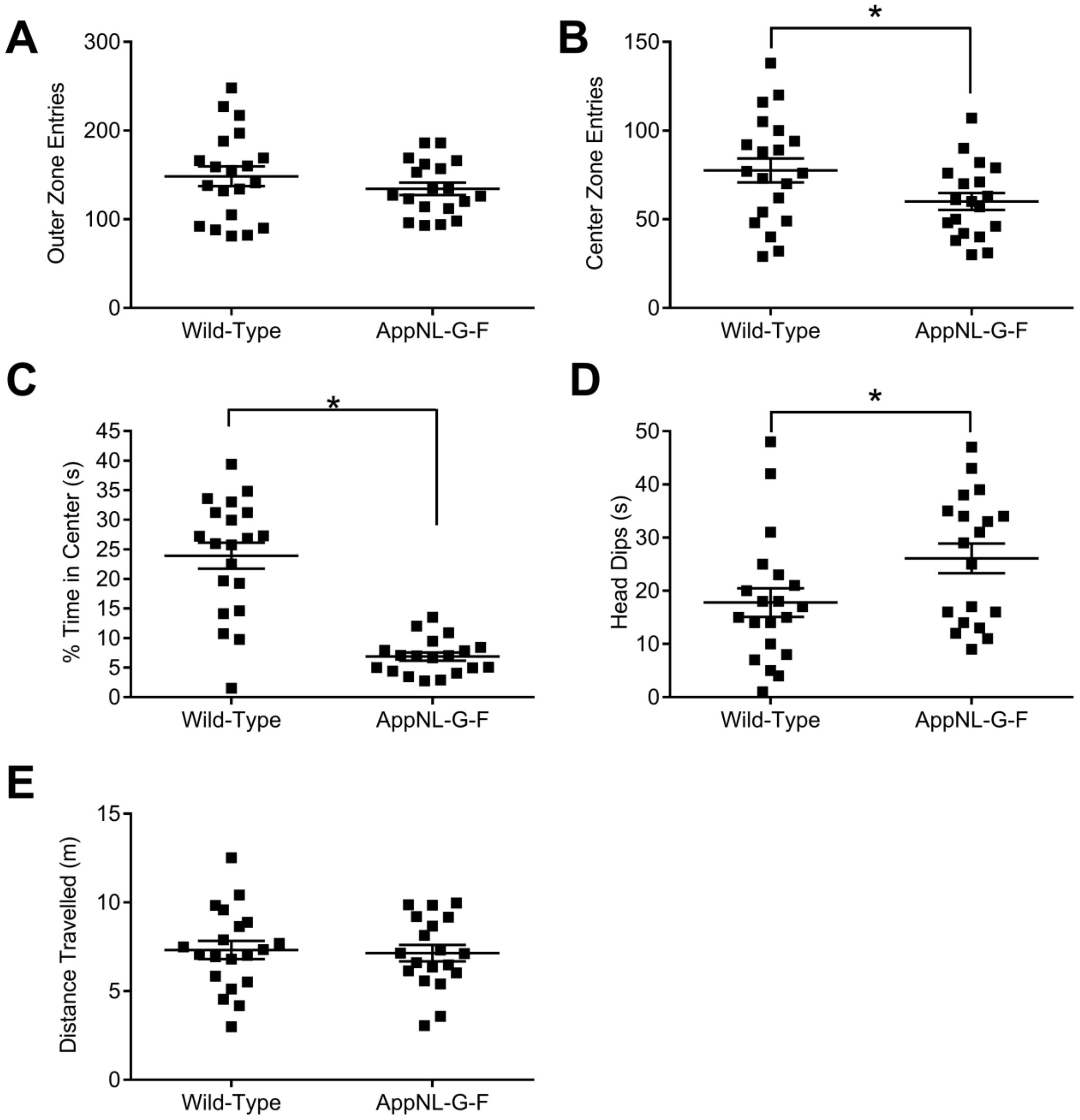

Figure S1. A. No significant differences were observed for entries made to the outer zone (genotype $F_{(1, 35)} = 1.43$, p = 0.240, genotype x sex $F_{(1, 35)} <1$, p = 0.928). **B.** *App*$^{NL\text{-}G\text{-}F}$ KI mice made fewer entries into the centre zone (genotype $F_{(1, 35)} = 5.12$, p = 0.030, genotype x sex $F_{(1, 35)} <1$, p = 0.727). **C.** *App*$^{NL\text{-}G\text{-}F}$ KI mice spent significantly less time in the centre zone in the elevated plus maze (genotype $F_{(1, 35)} = 5.39$, p = 0.030, genotype x sex $F_{(1, 35)} <1$, p = 0.850). **D.** There was a main effect of genotype for the number of head dips made in the elevated plus maze (genotype $F_{(1, 35)} = 5.13$, p = 0.030, genotype x sex $F_{(1, 35)} <1$, p = 0.577). **E.** Distance travelled in the elevated plus maze was similar between the genotypes (genotype $F_{(1, 35)} <1$, p = 0.861, genotype x sex $F_{(1, 35)} <1$, p = 0. 400).

Disease Models & Mechanisms • Supplementary information

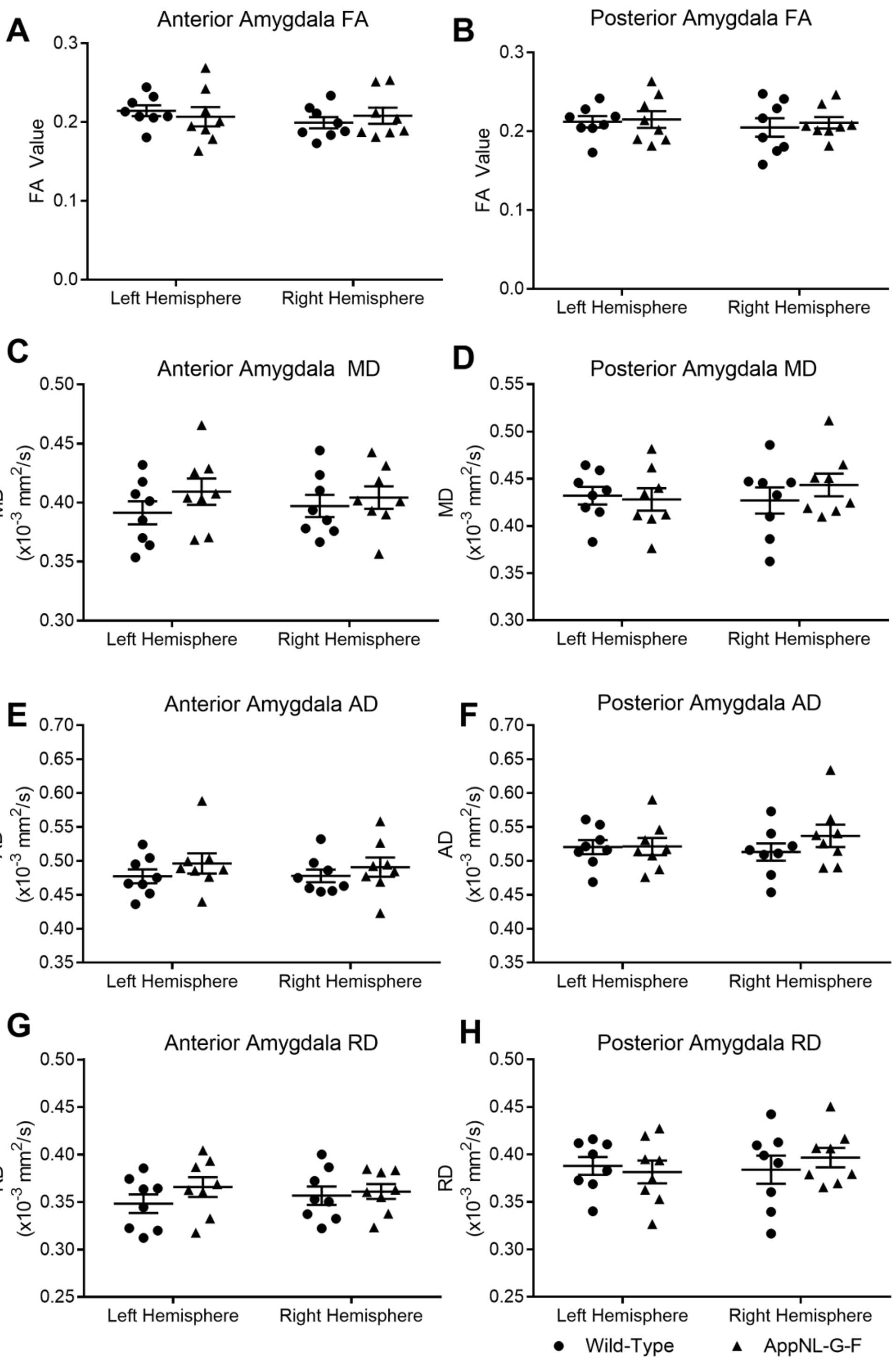

Figure S2. Fractional anisotropy (FA), mean diffusivity (MD), axial diffusivity (AD) and radial diffusivity (RD) in the amygdala. The entire amygdala was segmented from DTI images at two regions; anterior (**A, C, E, G**: Bregma -1.94 mm) and posterior (**B, D, F, H**: Bregma -2.46 mm). No significant differences were observed between the genotypes for anterior or posterior regions. Error bars represent s.e.m. Wild-type n=8, *App*$^{NL\text{-}G\text{-}F}$ KI n=8.

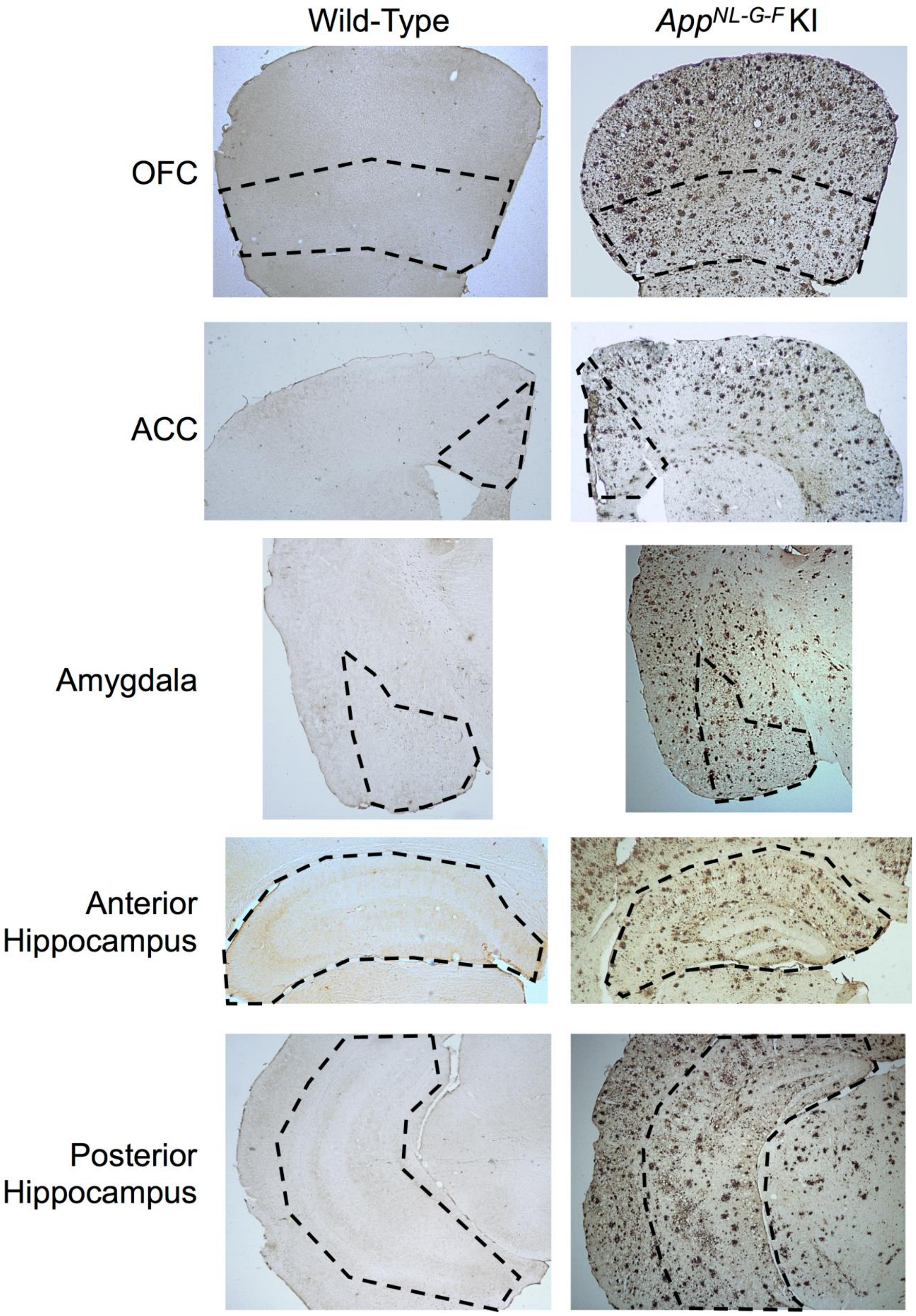

Figure S3. Amyloid plaque staining in wild-type and $App^{NL\text{-}G\text{-}F}$ KI mice in the orbitofrontal cortex (OFC), the anterior cingulate cortex (ACC) the amygdala, and the anterior and posterior hippocampus. Dashed line represents the brain regions of interest.
